# Measuring Pathological and Nonpathological Orthorexic Behavior: Validation of the Teruel Orthorexia Scale (TOS) among Polish Adults

**DOI:** 10.3390/nu16050638

**Published:** 2024-02-25

**Authors:** Wojciech Styk, Mateusz Gortat, Emilia Samardakiewicz-Kirol, Szymon Zmorzynski, Marzena Samardakiewicz

**Affiliations:** 1 Academic Laboratory of Psychological Tests, Medical University of Lublin, 20-093 Lublin, Poland; 2 Institute of Education and Practical Improvement, Association of Young Scientists in Poland, 20-560 Lublin, Poland; 3 Simulation Laboratory for Patient Safety, Department of Medical Education, Medical University of Lublin, 20-093 Lublin, Poland; 4 Laboratory of Genetics, Academy of Zamość, 22-400 Zamość, Poland; 5 Department of Psychology, Medical University of Lublin, 20-093 Lublin, Poland

**Keywords:** orthorexia nervosa, TOS, Polish adaptation of the teruel orthorexia scale, healthy orthorexia, pathological orthorexia

## Abstract

Measuring orthorexia nervosa is challenging due to the use of various existing tools and problems with sample representativeness. Another challenge for the Polish population is the adaptation of existing research tools and the evaluation of their relevance and research reliability. Our research aimed to adapt the TOS to the Polish language and measure pathological and nonpathological orthorexic behavior among the Polish population. The adaptation of the PL-TOS has high psychometric value and allows us to assess healthy and nervous orthorexia levels. This scale can be used not only for further research but also for diagnostic purposes in the daily work of clinicians and psychologists. Our results obtained in the present study indicate a correlation between TOS and both the use of supplements and diet. Higher TOS, ORTO_R and KZZJ_Diet Restrictions scores were obtained for individuals using dietary supplements than for those not using dietary supplements. In the future, it is worth conducting research aimed at various risk groups of individuals with orthorexia to confirm the psychometric properties of this adaptation of the TOS.

## 1. Introduction

Global nutritional trends have recently changed significantly. Increasingly, people are accessing food that has a beneficial effect on their health. In particular, more affluent people decide to choose products that are generally considered healthy [1]. Although these changes seem to be beneficial for public health, they may be associated with the risk of excessive interest in healthy eating, referred to as orthorexia nervosa (ON) [2].

The term orthorexia nervosa was described for the first time as an eating disorder by Bratman in 1997 [2]. It is difficult to establish a single coherent definition that would help to clearly define what orthorexia nervosa is. The most common factor is defined as too much attention given to choices related to restrictive eating, the avoidance of foods that are subjectively considered unhealthy and the elimination of specific groups of food such as meat, dairy, precooked food and frozen food or the elimination of salt, gluten, etc. The important factor is usually that the dietary regime has no basis in medical dietary recommendations. Another definition identifies orthorexia nervosa as “obsessive focus on dietary practices believed to promote optimum well-being through healthy eating (with inflexible dietary rules, recurrent and persistent preoccupations related to food, compulsive behaviors, etc.), with “consequent, clinically significant, impairment (e.g., medical or psychological complications, great distress, and/or impairment in important areas of functioning)” [3]. A person with ON can pay attention to the quality and origin of food, the form of storage and preparation of dishes, the way of eating meals and even the type of food packaging materials. This excessive concern about the nutritional process negatively affects patients’ functioning in society [3,4,5]. In extreme cases, orthorexia can lead to significant health deterioration, malnutrition or significant weight loss [6,7].

### 1.1. Epidemiology of Orthorexia Nervosa

The determinants of orthorexia nervosa have not been fully investigated. The literature suggests that psychological and sociocultural factors may play a significant role in its development [8]. The former may include an illusory sense of security (desire to prevent illness), the desire for total control over life (elimination of unpredictability), conformism (the theory associated with eating unconsciously allows one to achieve the culturally accepted model of a beautiful body), the search for spirituality and identity, and the desire to deprive one’s own emotional needs [9]. People with personality traits such as perfectionism, a high level of anxiety (tendency toward neophobia) and low self-esteem may have greater tendencies toward orthorexia [10,11,12,13]. Among the sociocultural factors, it is worth mentioning media promotion of a slim figure and the idea that being thin ensures happiness and success; the pursuit of certain professions (artists, athletes, medics and nutritionists); healthism; the perception of the slim body as a goal to achieve; the influence of social media and the Internet; the accessibility of organic food; social trends to eat healthy; dietary eating behaviors (vegetarianism and its variants); and religious or worldview practices [6,9,10,14,15,16]. Researchers do not agree on whether factors such as sex or self-esteem predispose individuals to the occurrence of orthorexia [15,17,18,19]. There is currently no evidence for a genetic determinant of orthorexia or its possible inheritance. Orthorexia nervosa can occur in patients with chronic illnesses, where diet is one of the important factors in taking care of their condition. However, the scientific evidence supporting such cases is mostly case-by-case descriptions, which makes generalization difficult [9].

It is difficult to determine a clear indication of the scale of incidence of orthorexia nervosa. Another recognized problem is the reliability of tools for evaluating ON. Orthorexia nervosa can be evaluated with the use of dedicated scales such as the ORTO-15, Düsseldorf Orthorexia Scale (DOS), Bratman Test for Orthorexia (BOT), Eating Habits Questionnaire (EHQ) and Teruel Orthorexia Scale (TOS). Unfortunately, those scales do not support clear diagnostic criteria. The problem is the unrepresentative study population in the majority of studies, which are usually conducted among populations at risk of developing orthorexia nervosa (vegetarians, artists, medics, etc.) [9].

Sources of data and scales of occurrence vary, ranging from 1% to 58% in the general population and even up to 90% in the at-risk population [9,17]. Significant variation in numbers may be due to cultural reasons or problems with the measurement and diagnosis of ON [7,9]. Orthorexia nervosa is often recognized at endocrine and metabolic clinics. A study in Turkey noted that among diabetes patients, orthorexia nervosa was more common in males (15.5%) than in females (11.1%). A higher incidence of orthorexia nervosa was also reported among obese patients [19,20].

### 1.2. Clinical Perspective

From a clinical perspective, there are no clear diagnostic criteria for orthorexia nervosa. There is still ongoing debate as to whether ON should be classified as a behavioral/lifestyle phenomenon or a mental disorder [21]. Currently, it is not classified as a clinical disease and is not included in the DSM-V (Diagnostic and Statistical Manual of Mental Disorders) or ICD-10 (International Statistical Classification of Diseases and Related Health Problems) diagnostic criteria for psychopathological disorders such as eating disorders or obsessive–compulsive disorders. At the end of 2022, a standardized definition of ON based on a worldwide, multidisciplinary cohort of experts was released. It summarizes observations, clinical knowledge and research findings. Although the participants span multiple countries and disciplines, further research will be needed to determine whether these diagnostic criteria are applicable to the experience of ON in geographic areas that are not represented by the current expert panel [22].

Research shows that patients with ON may have lower levels of well-being and life satisfaction, as well as greater levels of stress, than healthy individuals [21].

Orthorexia nervosa has a bidirectional relationship with other diseases. It is a disease that can be caused by the factors mentioned above, and the result of prolonged action can be endocrine and metabolic problems. On the other hand, endocrinopathy and treatment of endocrine diseases may cause anorexia nervosa. Orthorexia nervosa may cause endocrinopathy and, as a result, menstrual disturbance in women, osteoporosis and osteomalacia, which are the results of vitamin D deficiency and electrolyte imbalance. Moreover, the management of ON may cause endocrinopathy; for example, antipsychotic-induced dysmetabolism may occur. On the other hand, endocrinopathy may cause ON, which is associated with hypothalamic dysfunction and altered taste and conditions among diabetes patients. There are also situations in which the management of ON may involve coping with self-managing endocrinopathy or in which the management of endocrinopathy may precipitate ON. The first factor could be obesity and associated conditions or anorexia nervosa. In the second situation, a causative factor may be unsupervised weight loss programs [7].

Some behaviors, such as the excessive avoidance of salt, cereals or carbohydrates and the overconsumption of fruits, artificial sweeteners or proteins, are examples of malpractice resulting from a desire to take care of one’s health and from the use of misleading information sources [7]. These practices may also lead to endocrine problems.

Research into orthorexia has been ongoing for more than 20 years. The number of publications on orthorexia are systematically increasing. Researchers most frequently use the ORTO-15 test to estimate the degree of orthorexia. However, an increasing number of studies are accusing this test of overdiagnosing orthorexia, which has a low psychometric value [23,24,25,26]. The ORTO-15 scale has also been criticized for insufficient internal consistency and fluctuations in the Cronbach’s alpha value [27,28]. The doubts about the ORTO-15 scale prompted the authors to revise the scale. The revised ORTO-15 scale is named ORTO-R. The ORTO-R appears to be a valid alternative capable of overcoming such difficulties, but additional research is needed to confirm this [29].

In 2018, two Spanish researchers, Barrada and Roncero, developed the TOS (Teruel Orthorexia Scale) [30]. According to the authors’ assumptions, this questionnaire allows us to distinguish pathological orthorexia (unhealthy orthorexia) from healthy orthorexia, defined as an interest in healthy eating free from psychopathology. Moreover, it allows us to assess the relationship between other psychological constructs and disorders theoretically related to orthorexia, such as eating disorder symptoms, obsessive–compulsive disorders, negative affect and perfectionism [30,31]. The TOS consists of 17 items that can be answered on a four-point scale (from 1: “I do not agree at all” to 4: “I entirely agree”). Currently, the TOS questionnaire has been translated into German [32] (Strahler et al., 2020), Portuguese [33], Arabic [34] and English [35]. Despite the promising properties of this questionnaire, future research confirming its psychometric usefulness is needed [31].

The aim of this study was to translate and validate the TOS. It is the only two-dimensional scale that includes healthy and unhealthy orthorexia patients. Scale adaptations are important not only from a clinical perspective but also from a cross-cultural research perspective. Adopting tools for research in different cultures makes it possible to compare behaviors and measures of the same phenomena. The need for such studies and adaptations is also supported by other adaptations of tools examining ON, such as the PL-DOS and EHQ scales. The second goal was to investigate the properties of the scale by comparing it with existing scales like ORTO-R or TOS and the ON construct in the Polish population. We wanted to verify the co-occurrence of variables describing orthorexic behavior with other measures that are associated with eating disorders. We were also interested in examining whether these variables differentiated participants in terms of eating behaviors such as the use of diets or supplements.

## 2. Participants and Procedure

The study was approved by the Bioethics Committee at the Medical University of Lublin (consent no. KE-0254/223/2019). The data were collected using self-reported measures. The survey was administered at universities and workplaces. The questionnaire was anonymous, and the respondents completed it on their own.

A total of 713 people participated in the survey. After verification, 680 (80.3% female) questionnaires were included in further analysis. Thirty-three surveys were excluded due to the age of the respondents (<18) or lack of answers. The survey also collected sociodemographic and anthropometric data, such as weight and height, use of supplements and use of food.

## 3. Methods

**The Teruel Orthorexia Scale** by Barrada and Roncero (TOS) was used for the research, which, with the consent of the authors, was translated from Spanish into Polish by a translator, after which another translator performed a back-translation. After consulting a psychologist, the final version was established after comparing both versions. The questionnaire was validated using the guidelines included in the document describing the translation and adaptation of WHO instruments [36,37]. The Polish adaptation of the TOS consists of 17 statements. Individual questions on the TOS can be answered on a 5-point scale where 1 means the statement “does not concern you at all”, 5 means the statement “completely affects you” and 3 means a neutral attitude. The original version includes a 4-point response scale [30]. When adapting the scale to Polish, it was decided to expand the scale of responses by the so-called midpoint (neutral answer). The respondents were asked to use a 4-point scale to make an unambiguous declaration. Some researchers criticize the 4-point scale and recommend using a 5-point scale, which increases the reliability of the measurement [38,39]. The original scale was divided into two subscales: TOS He (nine items), healthy orthorexia without a pathological carrier; and TOS Ne (eight items), pathological/negative orthorexia. A higher score on both scales indicates a greater intensity of a given form of orthorexia. On the TOS Ne scale, position 10 was inverted. This result was recorded for the needs of the analyses. Due to the theoretical discrepancy between the two subscales, the overall score was calculated separately for TOS He and TOS Ne. The lowest possible result obtained for TOS He was nine and for TOS Ne, it was eight. The highest possible result in the TOS He subscale was 45, whereas that in the TOS Ne subscale was 40.

**The ORTO-R** questionnaire in the Polish adaptation [29] consists of 6 questions. The answers can be given on a 5-point scale. The ORTO-R scale is a revised version of the ORTO-15 questionnaire, and the revised version has better psychometric properties. Moreover, a univariate structure with good reliability has been confirmed in large sample studies [40].

**The KZZJ (Polish: Kwestionariusz Zachowań Związanych z Jedzeniem)** questionnaire is an eating behavior scale developed by Ogińska-Bulik and Putyński [41]. The KZZJ was developed based on the Eating Disorder Inventory and the Eating Attitude Test. The KZZJ consists of 30 items that make up the 3 scales of the questionnaire: Habitual Overeating (KZZJ_HO), Emotional Overeating (KZZJ_EO) and Diet Restrictions (KZZJ_DR). The test allows us to examine the tendency to overeat or refrain from eating. It is recommended for assessing the risk of overweight or obesity in normal-weight individuals and for observing progress during obesity treatment. According to the authors, the NRS can be used across age groups in obese and nonobese individuals and those with eating disorders.

**The Body Esteem Scale** (BES) by S. Franzoi and S. Shields, adapted in Polish by M. Lipowska and M. Lipowski, consists of 35 test items in three subscales. The subscales for women are AS—sexual attractiveness; KW—weight control; KF—physical conditioning. The subscales for men are AF—physical attractiveness; SC—body strength; KF—physical conditioning. To analyze the results of this study, we used the overall scale score without analyzing the subscale scores.

**Body mass index** (BMI) is a value derived from the mass (weight) and height of a person. BMI is defined as the body mass divided by the square of the body height and is expressed in units of kg/m^2^. The major adult BMI classifications are underweight (under 18.5 kg/m^2^), normal weight (18.5 to 24.9), overweight (25 to 29.9), and obese (30 or more) [42]. The subjects’ BMI was calculated based on their declared height and weight.

## 4. Results

The first step was to examine whether the collected dataset was suitable for conducting a factor analysis. For this purpose, the Kaiser–Meyer–Olkin test and Bartlett’s sphericity test were applied. The analyses indicated the adequacy of the collected data (χ2 = 6999.13; *p* < 0.001; KMO > 0.7). In the next step, a factor analysis was conducted, which confirmed the structure consists of two factors. The rotated component analysis identified item number 10 from the original scale as not sufficiently loading any of the factors. The other items were loaded as in the original scale. The loadings of each item are shown in Table 1. The lack of fit of item number 10 to the tested model was also confirmed by a reliability analysis performed using Cronbach’s alpha. The analysis indicated an improvement in Cronbach’s alpha for the subscale, but only after discarding item no. 10. Therefore, it was decided not to include item no. 10 in the final version of the scale. As a result, the final version obtained for the TOS_He scale was α_Cr_ = 0.87, and for the TOS Ne scale, α_Cr_ = 0.82. Confirmatory factor analysis performed in the final step confirmed sufficient model fit for the Polish scale without item no. 10 (RMSEA = 0.07; GFI = 0.95). The psychometric properties presented indicate comparable psychometric properties with those of the original scale. The final version of the Polish TOS, along with the STEN norms established in our sample, can be found in Appendix A.

## 5. Analysis of the Properties of the Polish TOS

**Figure 1:**
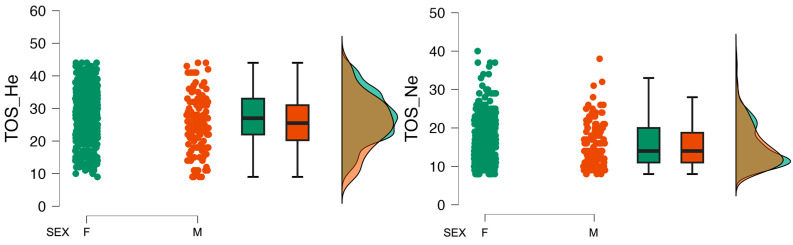
Distribution of TOS variables by sex.

The collected data were analyzed for differences in mean values across sex groups. Statistically significant differences were observed for the variables describing orthorexia behaviors: TOS_He and ORTO_R. Differences were also observed for variables describing diet restrictions, body image and BMI. The Cohen’s *d* effect size for these differences should be classified as small. A comparison across sex groups is shown in Table 2, and the distributions of TOS variables by sex are shown in Figure 1.

The results were also analyzed in terms of dietary supplement use. Significantly higher scores on the TOS, the ORTO_R scale and the KZZJ_Diet Restrictions scale were obtained by respondents who used supplements than by those who did not use supplements. The highest strength of this effect was observed for TOS_He (Cohen’s d = 0.49). These relationships are shown in Table 3.

We also checked whether the variables obtained using the Polish TOS differed according to body weight normality and diet. Analyses showed no differences in the mean values of TOS_He or TOS_Ne according to the BMI criterion between the groups for normal body weight. In contrast, comparisons of these variables among the groups of participants who were on a diet, had been on a diet or had never been on a diet indicated statistically significant differences in the values obtained in these groups. The strength of the effect of these differences was proven to be average (η^2^ = 0.08). The results of these comparisons are presented in Table 4.

**Figure 2:**
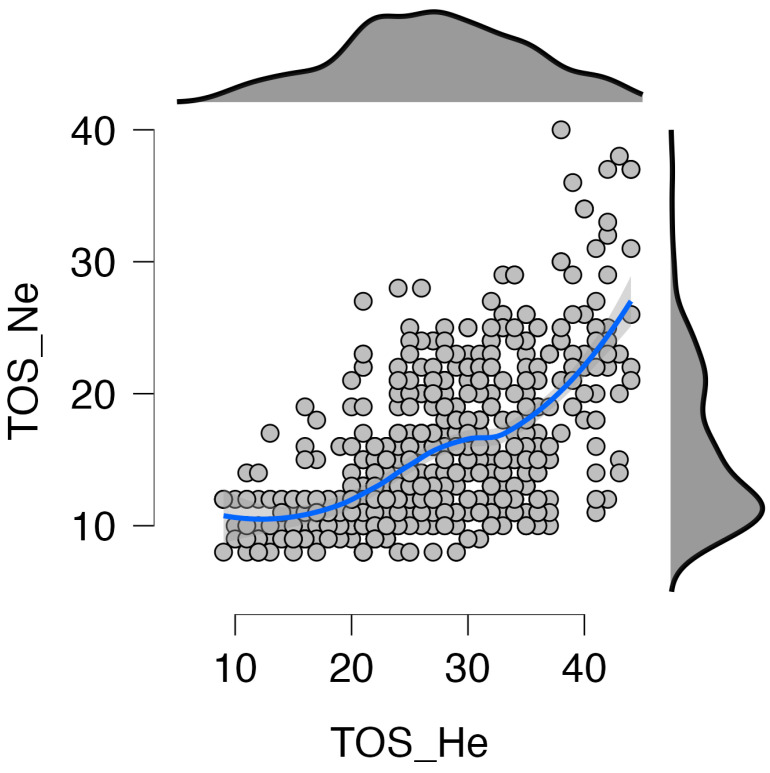
TOS subscale scatterplot and distribution. Blue line indicates correlations of scales, gray dots represent analyzed cases.

For the collected data, relationships between variables were examined using Pearson correlation. The results are shown in Table 5 and Figure 2. An important correlation was observed between the two scales, TOS and ORTO_R. The strength of the effect of these relationships was found to be medium. KZZJ_DR was significantly correlated with TOS_He (r = 0.25) and TOS_Ne (r = 0.49). In the next step, Fisher’s Z test was performed. The correlations of KZZJ_DR with TOS_He and KZZJ_DR with TOS_Ne were significantly different (Z = 9.805; *p* < 0.01). A stronger relationship with dietary restrictions was observed for the TOS_Ne subscale than for the TOS_He subscale.

## 6. Discussion

The obtained results allowed us to consider the Polish adaptation of the TOS questionnaire a reliable scale for identifying the phenomenon of orthorexia in the adult population. All of the items achieved satisfactory discriminant power. Factor analysis of the TOS in Polish confirmed that the structure consists of two factors: TOS He—healthy orthorexia and TOS Ne—pathological/negative orthorexia. Both of these factors showed good reliability. The Cronbach’s alpha coefficient was 0.87 for TOS He and 0.78 for TOS Ne. The obtained reliability is comparable to that of the original scale and other adaptations [43,44]. The authors of the Polish version decided to use a five-point scale to enable the respondents to declare a “neutral” relationship, which, as some studies indicate, increases the reliability of the obtained results [39].

The rotated component analysis identified item number 10 from the original scale as not sufficiently loading any of the factors. This is why this item was removed from the scale. Other adaptations of this scale also encountered difficulties in loading individual items. Like us, the authors of other adaptations decided to remove items [43]. This procedure did not negatively affect the quality of the validation.

Analysis of mean values across sex groups revealed statistically significant differences in orthorexic behaviors: TOS_He and ORTO_R had a small Cohen’s coefficient for diet restrictions, body image and BMI. Research on ON and its association with sex has yielded mixed results. Hallit found that females had greater ON tendencies than males, while Dell’Osso reported greater ON symptoms in females and those with high autistic traits [45,46]. Research has shown a strong correlation between orthorexia nervosa and body image, particularly in relation to perfectionism, appearance orientation and overweight preoccupation [10]. This correlation is particularly strong for female university students, where high body area satisfaction, low fitness orientation, low overweight preoccupation and low appearance orientation are predictors of orthorexia nervosa [47]. However, this relationship is not as clear in male students, where aspects of body image are not associated with orthorexic behaviors. Further research is needed to better understand the causal relationships and gender differences involved [40,45,47].

The most important results obtained in this study indicate a correlation between TOS and both the use of supplements and diet. Higher TOS, ORTO_R and KZZJ_Diet Restrictions scores were obtained for individuals using dietary supplements than for those not using dietary supplements. Orthorexia, an obsessive focus on healthy eating, is a growing concern in the field of nutrition and eating disorders [6]. It is often associated with the use of supplements and a restrictive diet, leading to social isolation and potential health risks [28]. The strongest relationship between dietary supplement usage and healthy orthorexia was noted. In conclusion, the use of dietary supplements may indicate a preoccupation with health and healthy eating. A significant correlation was also noted between both the TOS and dietary restrictions. A stronger relationship with dietary restrictions was observed for the TOS_Ne subscale than for the TOS_He subscale. This may lead to the conclusion that a strict dietary regimen may be a factor causing orthorexia nervosa.

Orthorexia, an obsessive focus on healthy eating, is associated with a range of factors. Bartrina noted that this practice often leads to a restrictive diet and social isolation [48]. Agopyan reported a high incidence of orthorexia in female nutrition and dietetics students, with a negative correlation between orthorexia and eating disorders [49]. A relationship between orthorexia and healthier food choices, as well as specific lifestyle habits, was identified [50]. Finally, Kaźmierczak-Wojtaś reported that individuals with orthorexic tendencies had a moderate intensity of healthy eating and a low intensity of unhealthy eating, but their nutritional knowledge did not significantly differ from that of other groups [51]. Both our study and others collectively demonstrate the need for a multidimensional approach to ON. At the forefront is the need to develop diagnostic criteria and tools, as well as strategies for primary and secondary prevention.

The main limitation of research on ON, and therefore of our study, is the lack of consistent diagnostic criteria. In recent years, work on orthorexia has been conducted at various research centers around the world. Currently, the proposed diagnostic criteria differ slightly from each other but are still not unambiguous. Further research is needed to help unify these findings and identify the right tools to capture the broad dimensions of this phenomenon. Since there are no official diagnostic criteria for ON, it was impossible to determine which participants had ON and treat them as a clinical group. In addition, we did not control our participants for any comorbidities other than ON. However, ON is not an official diagnosis, and there are no patient databases or other official records from which a representative sample can be obtained. Therefore, these results may not be representative of all individuals with ON.

## 7. Conclusions

The TOS is an important alternative to the ORTO-15 and ORTO-R scales. The TOS extends the diagnosis of orthorexia by two factors: healthy orthorexia and nervous orthorexia. Therefore, this scale may allow for a better understanding of the psychological aspects of orthorexia. The Polish version of the TOS is relatively short and characterized by internal consistency, validity and reliability.

## Figures and Tables

**Table 1:** Exploratory factor analysis.

	He	Ne
Czuje się w zgodzie ze sobą, kiedy odżywiam się zdrowo. (I feel good when I eat healthy food)	0.68	
2. Spędzam wiele czasu kupując, planując i/lub przygotowując jedzenie by moja dieta była tak zdrowa jak to możliwe. (I spend a lot of time buying, planning and/or preparing food so my diet will be as healthy as possible)	0.70	
3. Wierzę, że jadam zdrowiej niż większość ludzi. (I believe that the way I eat is healthier than that of most people)	0.77	
4. Obwiniam się, gdy jem rzeczy postrzegane jako niezdrowe. (I feel guilty when I eat food that I do not consider healthy)		0.80
5. Moje przekonania dotyczące zdrowego jedzenia negatywnie wpłynęły na moje relacje z innymi. (My social relations have been negatively affected by my concern about eating healthy food)		0.64
6. Moje zainteresowanie zdrową żywnością jest ważną częścią tego kim jestem i jak rozumiem świat. (My interest in healthy food is an important part of the way I am, of how I understand the world)	0.84	
7. Preferuje bardziej zjedzenie zdrowego posiłku, który nie jest bardzo smaczny, niż smacznego posiłku, który jest niezdrowy. (I’d rather eat a healthy food that is not very tasty than a good tasting food that is not Healthy)	0.64	
8. Głównie jem żywność, którą postrzegam za zdrową. (I mainly eat foods that I consider to be healthy)	0.80	
9. Poświęcam wiele czasu na szukanie informacji o zdrowej żywności. (I am concerned about the possibility of eating unhealthy foods)		0.78
10. Martwi mnie możliwość jedzenia niezdrowych rzeczy. (I am concerned about the possibility of eating unhealthy foods)		0.01
11. Nie przeszkadza mi wydawanie dużej ilości pieniędzy na produkty, które postrzegam za bardziej zdrowe od innych. (I do not mind spending more money on food if I think it is healthier)	0.51	
12. Czuje się przygnębiony i smutny, jeśli zjem coś niezdrowego. (I feel overwhelmed or sad if I eat food that I consider unhealthy)		0.82
13. Wolę raczej zjeść małą porcję zdrowego posiłku niż dużą porcję posiłku, który może być niezdrowy. (I prefer to eat a small quantity of healthy food rather than a lot of food that may not be healthy)	0.70	
14. Unikam jedzenie z osobami, które żywią się niezdrowo. (I avoid eating with people who do not share my ideas about healthy eating)		0.62
15. Staram się przekonać osoby w moim otoczeniu do podążania za moimi nawykami żywieniowymi. (I try to convince people from my environment to follow my healthy eating habits)	0.67	
16. Obwiniam siebie, gdy zjem coś, co postrzegam jako niezdrowe. (If, at some point, I eat something that I consider unhealthy, I punish myself for it)		0.85
17. Ciągle myślenie o zdrowym jedzeniu nie pozwala mi skoncentrować się na wykonywaniu innych zadań. (Thoughts about healthy eating do not let me concentrate on other tasks)		0.75

Note. Applied rotation method is promax.

**Table 2:** Comparison of sex groups.

	SEX	N	Mean	SD	*p*	Cohen’s d
TOS_He	F	546	27.48	7.57	<0.001	0.26
	M	134	25.49	8.37		
TOS_Ne	F	546	15.48	5.75	0.67	
	M	134	15.25	5.61		
ORTO_R	F	546	13.90	3.09	<0.01	0.28
	M	134	13.02	3.55		
KZZJ_HO	F	546	3.98	2.80	0.69	
	M	134	3.87	2.57		
KZZJ_EO	F	546	4.46	2.43	0.79	
	M	134	4.52	2.61		
KZZJ_DR	F	546	3.44	2.60	0.05	0.19
	M	134	2.95	2.28		
BES	F	546	229.54	50.43	0.03	0.21
	M	134	240.59	58.01		
BMI	F	546	22.58	4.12	<0.01	0.27
	M	134	23.70	3.95		
Age	F	546	28.27	10.06	0.16	
	M	134	26.94	8.37		

**Table 3:** Comparison of supplement use groups.

	Sup *	N	Mean	SD	*p*	Cohen’s d
TOS_He	No	259	24.78	7.67	<0.001	0.49
	Yes	421	28.51	7.49		
TOS_Ne	No	259	14.17	4.66	<0.001	0.36
	Yes	421	16.21	6.16		
ORTO_R	No	259	13.14	3.18	<0.001	0.30
	Yes	421	14.09	3.16		
KZZJ_HO	No	259	4.14	2.90	0.18	
	Yes	421	3.85	2.66		
KZZJ_EO	No	259	4.35	2.46	0.32	
	Yes	421	4.54	2.47		
KZZJ_DR	No	259	2.92	2.30	<0.001	0.27
	Yes	421	3.60	2.65		
BES	No	259	229.60	52.32	0.41	
	Yes	421	233.02	52.07		
BMI	No	259	23.03	4.36	0.09	
	Yes	421	22.66	3.94		
Age	No	259	27.85	9.58	0.76	
	Yes	421	28.10	9.87		

* Use of nutritional supplements.

**Table 4:** Comparison of TOS_He and TOS_Ne among the different diet use groups.

		N	Mean	SD	Comparison	*p*
TOS_He	1. I’m on a diet	71	31.07	7.69	1–2	<0.001
	2. I have been on a diet	371	27.59	7.36	1–3	<0.001
	3. I have never been on a diet	238	25.12	7.88	2–3	<0.001
TOS_Ne	1. I’m on a diet	71	18.97	7.02	1–2	<0.001
	2. I have been on a diet	371	15.99	5.68	1–3	<0.001
	3. I have never been on a diet	238	13.51	4.57	2–3	<0.001

**Table 5:** Pearson’s correlation.

		TOS_He	TOS_Ne	ORTO-R	BES	KZZJ_HO	KZZJ_EO	BMI	AGE
TOS_Ne	Pearson’s r	0.58 ***	—						
	Effect size (Fisher’s z)	0.66	—						
ORTO_R	Pearson’s r	0.43 ***	0.46 ***	—					
	Effect size (Fisher’s z)	0.46	0.50	—					
BES	Pearson’s r	0.13 ***	−0.08 *	−0.21 ***	—				
	Effect size (Fisher’s z)	0.13	−0.08	−0.22	—				
KZZJ_HO	Pearson’s r	−0.06	0.16 ***	0.35 ***	−0.25 ***	—			
	Effect size (Fisher’s z)	−0.06	0.16	0.36	−0.26	—			
KZZJ_EO	Pearson’s r	0.03	0.32 ***	0.37 ***	−0.35 ***	0.55 ***	—		
	Effect size (Fisher’s z)	0.03	0.33	0.39	−0.37	0.62	—		
KZZJ_DR	Pearson’s r	0.25 ***	0.49 ***	0.45 ***	−0.33 ***	0.24 ***	0.49 ***	—	
	Effect size (Fisher’s z)	0.26	0.53	0.48	−0.34	0.24	0.54	—	
BMI	Pearson’s r	−0.09 *	−0.04	−0.02	−0.19 ***	0.07	0.16 ***	0.17 ***	—
	Effect size (Fisher’s z)	−0.09	−0.04	−0.02	−0.19	0.07	0.16	0.17	—
Age	Pearson’s r	0.11 **	−0.04	−0.10 *	0.01	−0.19 ***	−0.11 **	−0.03	0.37 ***
	Effect size (Fisher’s z)	0.11	−0.04	−0.10	0.01	−0.19	−0.11	−0.03	0.38

* *p* < 0.05; ** *p* < 0.01; *** *p* < 0.001.

## Data Availability

The data are contained within the article.

## Abbreviations

BES	Body Esteem Scale
BMI	Body Mass Index
KZZJ_HO	Habitual Overeating subscale
KZZJ_EO	Emotional Overeating subscale
KZZJ_DR	Diet Restrictions subscale
ON	Orthorexia Nervosa
TOS	Teruel Orthorexia Scale
TOS Ne	Subscale of the TOS examining the severity of orthorexia, referred to as nervous (pathological)
TOS He	Subscale of the TOS examining the severity of orthorexia, referred to as healthy (nonpathological)
HeOr	Orthorexia described as healthy (nonpathological)
OrNe	orthorexia defined as nervous (pathological)

## Appendix A

Teruel Orthorexia Scale (TOS) adapted in Polish by M. Gortat and M. Samardakiewicz						
Poniższe pytania odnoszą się do Twoich przekonań i zachowań dotyczących żywienia. Chcemy poznać jak ważne jest przestrzeganie zdrowej diety lub spożywanie pokarmów, pozbawionych tłuszczu, soli, konserwantów, dodatkowych substancji, które uważasz za szkodliwe lub toksyczne. Zaznacz w jakim stopniu zgadzasz się z poniższymi twierdzeniami. Jeśli zaznaczysz 1 będzie to oznaczać, że to twierdzenie zupełnie nie dotyczy Ciebie, jeśli zaznaczysz 5 oznacza to, że twierdzenie w pełni dotyczy Ciebie.						
1	Czuje się w zgodzie ze sobą, kiedy odżywiam się zdrowo.	1	2	3	4	5
2	Spędzam wiele czasu kupując, planując i/lub przygotowując jedzenie by moja dieta była tak zdrowa jak to możliwe.	1	2	3	4	5
3	Wierzę, że jadam zdrowiej niż większość ludzi.	1	2	3	4	5
4	Obwiniam się gdy jem rzeczy postrzegane jako niezdrowe.	1	2	3	4	5
5	Moje przekonania dotyczące zdrowego jedzenia negatywnie wpłynęły na moje relacje z innymi.	1	2	3	4	5
6	Moje zainteresowanie zdrową żywnością jest ważną częścią tego kim jestem i jak rozumiem świat.	1	2	3	4	5
7	Preferuje bardziej zjedzenie zdrowego posiłku, który nie jest bardzo smaczny, niż smacznego posiłku, który jest niezdrowy.	1	2	3	4	5
8	Głównie jem żywność, którą postrzegam za zdrową.	1	2	3	4	5
9	Poświęcam wiele czasu na szukanie informacji o zdrowej żywności.	1	2	3	4	5
10	Nie przeszkadza mi wydawanie dużej ilości pieniędzy na produkty, które postrzegam za bardziej zdrowe od innych.	1	2	3	4	5
11	Czuje się przygnębiony i smutny, jeśli zjem coś niezdrowego.	1	2	3	4	5
12	Wolę raczej zjeść małą porcję zdrowego posiłku niż dużą porcję posiłku, który może być niezdrowy.	1	2	3	4	5
13	Unikam jedzenie z osobami, które żywią się niezdrowo.	1	2	3	4	5
14	Staram się przekonać osoby w moim otoczeniu do podążania za moimi nawykami żywieniowymi.	1	2	3	4	5
15	Obwiniam siebie, gdy zjem coś, co postrzegam jako niezdrowe.	1	2	3	4	5
16	Ciągle myślenie o zdrowym jedzeniu nie pozwala mi skoncentrować się na wykonywaniu innych zadań.	1	2	3	4	5

**STEN norms**

TOS_He_STEN										
SEX	1	2	3	4	5	6	7	8	9	10
F	9–14	15–17	18–21	22–25	26–29	30–33	34–36	37–40	41–44	45
M	9–10	11–14	15–19	20–23	24–27	28–31	32–35	36–40	41–44	45
TOS_Ne_STEN										
SEX	1	2	3	4	5	6	7	8	9	10
F		7	8–10	11–13	14–16	17–18	19–21	22–24	25–27	28–35
M		7	8–10	11–12	13–15	16–18	19–21	22–24	25–27	28–35

No. item	Subscale TOS
1	He
2	He
3	He
4	Ne
5	Ne
6	He
7	He
8	He
9	Ne
10	He
11	Ne
12	He
13	Ne
14	He
15	Ne
16	Ne

## References

[1] Liu J., Micha R., Li Y., Mozaffarian D. (2021). Trends in Food Sources and Diet Quality among US Children and Adults, 2003–2018. JAMA Netw. Open.

[2] Bratman S. (2017). Orthorexia vs. Theories of Healthy Eating. Eat. Weight. Disord..

[3] Koven N., Abry A. (2015). The Clinical Basis of Orthorexia Nervosa: Emerging & Perspectives. Neuropsychiatr. Dis. Treat..

[4] Cena H., Barthels F., Cuzzolaro M., Bratman S., Brytek-Matera A., Dunn T., Varga M., Missbach B., Donini L.M. (2019). Definition and Diagnostic Criteria for Orthorexia Nervosa: A Narrative Review of the Literature.

[5] Hayatbini N., Oberle C.D. (2019). Are Orthorexia Nervosa Symptoms Associated with Cognitive Inflexibility?. Psychiatry Res..

[6] Hanganu-Bresch C. (2020). Orthorexia: Eating Right in the Context of Healthism. Med. Humanit..

[7] Kalra S., Kapoor N., Jacob J. (2020). Orthorexia Nervosa. J. Pak. Med. Assoc..

[8] Bóna E., Túry F., Forgács A. (2019). Evolutionary Aspects of a New Eating Disorder: Orthorexia Nervosa in the 21st Century. Psychol. Thought.

[9] Grajek M., Sas-Nowosielski K. (2022). Review of Available Diagnostic Options for Orthorexia Nervosa. J. Educ. Health Sport.

[10] Barnes M.A., Caltabiano M.L. (2017). The Interrelationship between Orthorexia Nervosa, Perfectionism, Body Image and Attachment Style. Eat. Weight. Disord..

[11] Novara C., Pardini S., Maggio E., Mattioli S., Piasentin S. (2021). Orthorexia Nervosa: Over Concern or Obsession about Healthy Food?. Eat. Weight. Disord..

[12] Brown A.J., Parman K.M., Rudat D.A., Craighead L.W. (2012). Disordered Eating, Perfectionism, and Food Rules. Eat. Behav..

[13] Bardone-Cone A.M., Wonderlich S.A., Frost R.O., Bulik C.M., Mitchell J.E., Uppala S., Simonich H. (2007). Perfectionism and Eating Disorders: Current Status and Future Directions. Clin. Psychol. Rev..

[14] Bóna E., Forgács A., Túry F. (2018). Potential Relationship between Juice Cleanse Diets and Eating Disorders. A Qualitative Pilot Study. Orv. Hetil..

[15] McComb S.E., Mills J.S. (2019). Orthorexia Nervosa: A Review of Psychosocial Risk Factors. Appetite.

[16] Ross Arguedas A.A. (2020). “Can Naughty Be Healthy?”: Healthism and Its Discontents in News Coverage of Orthorexia Nervosa. Soc. Sci. Med..

[17] Kaźmierczak-Wojtaś N., Patryn R., Zagaja A., Drozd M., Niedzielski A. (2021). Prevalence and Characteristics of Orthorectic Disorders in Adolescence and Young People: Polish Preliminary Studies. Nutrients.

[18] Malmborg J., Bremander A., Olsson M.C., Bergman S. (2017). Health Status, Physical Activity, and Orthorexia Nervosa: A Comparison between Exercise Science Students and Business Students. Appetite.

[19] Oberle C.D., Samaghabadi R.O., Hughes E.M. (2017). Orthorexia Nervosa: Assessment and Correlates with Gender, BMI, and Personality. Appetite.

[20] Dunn T.M., Gibbs J., Whitney N., Starosta A. (2017). Prevalence of Orthorexia Nervosa Is Less than 1%: Data from a US Sample. Eat. Weight. Disord. Stud. Anorex. Bulim. Obes..

[21] Pontillo M., Zanna V., Demaria F., Averna R., Di Vincenzo C., De Biase M., Di Luzio M., Foti B., Tata M.C., Vicari S. (2022). Orthorexia Nervosa, Eating Disorders, and Obsessive-Compulsive Disorder: A Selective Review of the Last Seven Years. J. Clin. Med..

[22] Donini L.M., Barrada J.R., Barthels F., Dunn T.M., Babeau C., Brytek-Matera A., Cena H., Cerolini S., Cho H., Coimbra M. (2022). A Consensus Document on Definition and Diagnostic Criteria for Orthorexia Nervosa. Eat. Weight. Disord..

[23] Almeida C., Borba V.V., Santos L. (2018). Orthorexia Nervosa in a Sample of Portuguese Fitness Participants. Eat. Weight. Disord. Stud. Anorex. Bulim. Obes..

[24] Gramaglia C., Brytek-Matera A., Rogoza R., Zeppegno P. (2017). Orthorexia and Anorexia Nervosa: Two Distinct Phenomena? A Cross-Cultural Comparison of Orthorexic Behaviours in Clinical and Non-Clinical Samples. BMC Psychiatry.

[25] Missbach B., Dunn T.M., König J.S. (2017). We Need New Tools to Assess Orthorexia Nervosa. A Commentary on “Prevalence of Orthorexia Nervosa among College Students Based on Bratman’s Test and Associated Tendencies”. Appetite.

[26] Reynolds R. (2018). Is the Prevalence of Orthorexia Nervosa in an Australian University Population 6.5%?. Eat. Weight Disord..

[27] Depa J., Barrada J., Roncero M. (2019). Are the Motives for Food Choices Different in Orthorexia Nervosa and Healthy Orthorexia?. Nutrients.

[28] Varga M., Thege B.K., Dukay-Szabó S., Túry F., van Furth E.F. (2014). When Eating Healthy Is Not Healthy: Orthorexia Nervosa and Its Measurement with the ORTO-15 in Hungary. BMC Psychiatry.

[29] Rogoza R., Donini L.M. (2021). Introducing ORTO-R: A Revision of ORTO-15. Eat. Weight. Disord. Stud. Anorex. Bulim. Obes..

[30] Barrada J.R., Roncero M. (2018). Bidimensional Structure of the Orthorexia: Development and Initial Validation of a New Instrument. An. Psicol..

[31] Valente M., Syurina E.V., Donini L.M. (2019). Shedding Light upon Various Tools to Assess Orthorexia Nervosa: A Critical Literature Review with a Systematic Search. Eat. Weight. Disord..

[32] Strahler J., Haddad C., Salameh P., Sacre H., Obeid S., Hallit S. (2020). Cross-Cultural Differences in Orthorexic Eating Behaviors: Associations with Personality Traits. Nutrition.

[33] Roberto da Silva W., Cruz Marmol C.H., Nogueira Neves A., Marôco J., Bonini Campos J.A.D. (2021). A Portuguese Adaptation of the Teruel Orthorexia Scale and a Test of Its Utility with Brazilian Young Adults. Percept. Mot. Skills.

[34] Mhanna M., Azzi R., Hallit S., Obeid S., Soufia M. (2021). Validation of the Arabic Version of the Teruel Orthorexia Scale (TOS) among Lebanese Adolescents. Eat. Weight Disord..

[35] Chace S., Kluck A.S. (2021). Validation of the Teruel Orthorexia Scale and Relationship to Health Anxiety in a U.S. Sample. Eat. Weight Disord. Stud. Anorex. Bulim. Obes..

[36] Gawlik M., Donata Kurpas F. (2014). Principles of Questionnaires’ Validation on the Example of the Caregiver Quality of Life-Cancer (CQOL-C) Questionnaire. Med. Sci. Pulse.

[37] World Health Organization Process of Translation and Adaptation of Instruments. http://www.who.int/substance_abuse/research_tools/translation/en/.

[38] Adelson J.L., McCoach D.B. (2010). Measuring the Mathematical Attitudes of Elementary Students: The Effects of a 4-Point or 5-Point Likert-Type Scale. Educ. Psychol. Meas..

[39] Chyung S.Y.Y., Roberts K., Swanson I., Hankinson A. (2017). Evidence-Based Survey Design: The Use of a Midpoint on the Likert Scale. Perform. Improv..

[40] Brytek-Matera A., Obeid S., Donini L.M., Rogoza M., Marchlewska M., Plichta M., Jezewska-Zychowicz M., Hallit S., Rogoza R. (2023). Psychometric Properties of the ORTO-R in a Community-Based Sample of Women and Men from Poland. J. Eat. Disord..

[41] Ogińska-Bulik N. (2004). The Psychology of Excessive Eating. Causes—Consequences—Ways to Change.

[42] Body Mass Index—BMI. http://www.euro.who.int/en/health-topics/disease-prevention/nutrition/a-healthy-lifestyle/body-mass-index-bmi.

[43] Lasson C., Rousseau A., Vicente S., Goutaudier N., Romo L., Roncero M., Barrada J.R. (2023). Orthorexic Eating Behaviors Are Not All Pathological: A French Validation of the Teruel Orthorexia Scale (TOS). J. Eat. Disord..

[44] Argyrides M., Anastasiades E., Maïano C., Swami V. (2023). Greek Adaptation of the Teruel Orthorexia Scale (TOS) in Adults from the Republic of Cyprus: A Bidimensional Model May Not Be Universal. Appetite.

[45] Hallit S., Brytek-Matera A., Obeid S. (2021). Orthorexia Nervosa and Disordered Eating Attitudes among Lebanese Adults: Assessing Psychometric Proprieties of the ORTO-R in a Population-Based Sample. PLoS ONE.

[46] Dell’Osso L., Carpita B., Muti D., Cremone I.M., Massimetti G., Diadema E., Gesi C., Carmassi C. (2018). Prevalence and Characteristics of Orthorexia Nervosa in a Sample of University Students in Italy. Eat. Weight Disord. Stud. Anorex. Bulim. Obes..

[47] Brytek-Matera A., Gramaglia C., Gambaro E., Delicato C., Zeppegno P. (2018). The Psychopathology of Body Image in Orthorexia Nervosa. J. Psychopathol..

[48] Bartrina J.A. (2007). Orthorexia or When a Healthy Diet Becomes an Obsession. Arch. Latinoam. Nutr..

[49] Agopyan A., Kenger E.B., Kermen S., Ulker M.T., Uzsoy M.A., Yetgin M.K. (2019). The Relationship between Orthorexia Nervosa and Body Composition in Female Students of the Nutrition and Dietetics Department. Eat. Weight Disord. Stud. Anorex. Bulim. Obes..

[50] Varga M., Dukay-Szabó S., Túry F., van Furth Eric F. (2013). Evidence and Gaps in the Literature on Orthorexia Nervosa. Eat. Weight Disord. Stud. Anorex. Bulim. Obes..

[51] Kujawowicz K., Mirończuk-Chodakowska I., Witkowska A.M. (2022). Dietary Behavior and Risk of Orthorexia in Women with Celiac Disease. Nutrients.

